# Dynamics of Passive and Active Particles in the Cell Nucleus

**DOI:** 10.1371/journal.pone.0045843

**Published:** 2012-10-15

**Authors:** Feroz M. Hameed, Madan Rao, G. V. Shivashankar

**Affiliations:** 1 Mechanobiology Institute, and Department of Biological Sciences, National University of Singapore, Singapore; 2 National Centre for Biological Sciences, Tata Institute of Fundamental Research, Bangalore, India; 3 Raman Research Institute, Bangalore, India; University of Massachusetts Medical, United States of America

## Abstract

Inspite of being embedded in a dense meshwork of nuclear chromatin, gene loci and large nuclear components are highly dynamic at 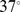
C. To understand this apparent unfettered movement in an overdense environment, we study the dynamics of a passive micron size bead in live cell nuclei at two different temperatures (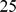 and 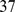 
C) with and without external force. In the absence of a force, the beads are caged over large time scales. On application of a *threshold* uniaxial force (about 10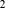 pN), the passive beads appear to hop between cages; this large scale movement is absent upon ATP-depletion, inhibition of chromatin remodeling enzymes and RNAi of lamin B1 proteins. Our results suggest that the nucleus behaves like an active solid with a finite yield stress when probed at a micron scale. Spatial analysis of histone fluorescence anisotropy (a measure of local chromatin compaction, defined as the volume fraction of tightly bound chromatin) shows that the bead movement correlates with regions of low chromatin compaction. This suggests that the physical mechanism of the observed yielding is the active opening of free-volume in the nuclear solid via chromatin remodeling. Enriched transcription sites at 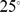
C also show caging in the absence of the applied force and directed movement beyond a yield stress, in striking contrast with the large scale movement of transcription loci at 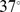
C in the absence of a force. This suggests that at physiological temperatures, the loci behave as *active* particles which remodel the nuclear mesh and reduce the local yield stress.

## Introduction

The 3D spatial assembly of nuclear chromatin is believed to be crucial for the specific patterning of in-vivo gene expression [1]. Biophysical measurements have shown that nuclear chromatin is organized as a heterogeneous mesh [2] with a typical mesh size of about 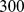
nm [3], and is dynamically remodeled by a variety of chromatin remodeling proteins [4]. To maintain this precise 3D architecture over interphase, mobile transcription elements should be able to move in a directed and regulated manner through this dense nuclear meshwork.

The dynamics of small molecules in the nucleus have been extensively studied using fluorescence methods [2], [5], [6], [7], [8], [9]; these microrheology studies suggest that at scales much smaller than the mesh size, the nucleus behaves as a viscoelastic fluid [10], [11]. On the other hand, studies on the dynamics of large nuclear bodies, though rather limited, reveal interesting temperature dependent behaviour. For instance, experiments on the dynamics of transcription compartments (TCs) [5], cajal bodies [6], PML bodies [7], and gene loci [12], have reported large scale directed movements at physiological temperatures, which are influenced by ATP-dependent chromatin remodeling, while similar studies on the dynamics of TCs at lower temperatures (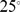
C) reveal a near absence of any large scale movement [5].

To understand the physical mechanisms underlying this temperature and scale dependent movement through the nucleus, we compare the dynamics of 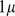
m size paramagnetic beads injected into the nucleus at two temperatures, viz., 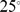
C and 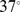
C, and contrast it to the dynamics of TCs at these temperatures. The beads, embedded in a network of protein and nucleic acid filaments which are actively driven and remodeled by a variety of molecular machines [13], [14], are thus subject to active stress fluctuations of the nuclear medium. These beads however do not actively remodel the chromatin; we will generically refer to such particles as being *passive particles*. On the other hand, nuclear bodies in conjunction with remodeling enzymes exert active stresses on the chromatin, at least at physiological temperatures; we will generically refer to them as *active particles*. This classification of probe particles, first introduced in the context of cell surface resident molecules [15], provides fresh insight into the rheology of the cell nucleus.

We find that in the absence of any applied force, large passive particles - viz., beads at 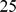 and 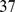 
C and TCs at 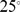
C - are essentially caged by the nuclear mesh. Microrheology of these large passive particles suggests that the nucleus responds more as an elastic medium at the lowest frequencies. On applying a force beyond a threshold (about 10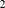 pN), the beads move roughly in the direction of the force - the trajectories appear as a series of hops between caged movements - and is absent under conditions of ATP-depletion or perturbations of chromatin remodeling proteins. The nucleus thus behaves like an active solid with a finite yield stress when probed at a micron scale. We argue that the external force on the embedded bead deforms the chromatin meshwork, this distortion in turn modulates the local volume fraction of the histone-bound chromatin which we monitor by histone fluorescence anisotropy. Spatial analysis of histone anisotropy is therefore a measure of local chromatin compaction (or chromatin condensation). We use fluorescence live-cell imaging to simultaneously map chromatin condensation and the dynamics of beads and TCs. Beyond the yield stress, the passive particle movement correlates with regions of low chromatin compaction. This suggests that the physical mechanism of the observed yielding is the active opening of free-volume in the nuclear solid, via ATP-dependent chromatin remodeling proteins. While the TCs behave as passive particles at 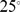
C, their behaviour is quite different at 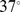
C, where we see large scale movements *even in the absence of a force* (this was reported earlier in [5]). This suggests that at physiological temperatures, the TCs behave as *active* particles, which move by actively remodeling the nuclear mesh to reduce the local yield stress.

## Materials and Methods

### Cell culture and chemical perturbations

Wild type HeLa cells and HeLa cells stably transfected with H2B-EGFP were grown in DMEM (Invitrogen Corporation, USA) supplemented with 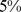 FBS (Invitrogen Corporation, USA) at 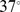
C and 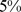 CO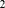. ATP depletion was done by incubation with 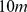
M Sodium azide and 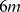
M 2-deoxy-D-Glucose in M1 buffer (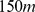
M NaCl, 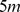
M KCl, 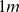
M CaCl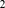, 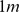
M MgCl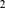, 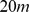
M HEPES, pH 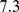) in the absence of glucose for 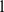 hour as has been optimised earlier in our laboratory [16]. Chromatin hyperacetylation was induced by incubation in 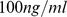 solution of Trichostatin A (TSA; Sigma, USA) in DMEM supplemented with 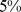 FBS at 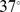
C for 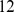 hours. Chromatin condensation was induced by treatment with 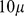
M Staurosporine in M1 buffer at 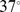
C for 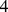 hours. Depletion of Lamin B1 was achieved by transfection with 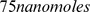 of siRNA (Lamin B1 sense siRNA - 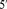
CGCGC UUGGU AGAGG UGGAT T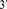, Lamin B1 antisense siRNA - 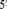
UCCAC CUCUA CCAAG CGCGT T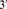, scrambled control sense RNA - 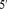
GAGGA CUGUC AUGGA GUGAT T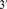, scrambled control antisense RNA - 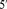
UCACU CCAUG ACAGU CCUCT T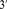) per 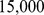 cells using DharmaFECT 1 Transfection reagent (Dharmacon RNA Technologies, USA) [2]. Cells were transfected at 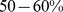 confluency in coverslip-bottomed 35 mm plates with 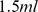 of media. Under these conditions Lamin B1 decreased by 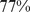 after 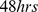 [2], [31]. Cells treated with the Lamin B1 siRNA were used after 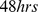 of transfection.

### Experimental Setup

The experimental setup consists of a custom built electromagnet arrangement mounted on a fluorescence microscope (model IX81; Olympus, Japan) with fluorescence anisotropy capability through split imaging on the chip of a cooled EMCCD camera (model iXon DV-887BV; Andor corp., USA). The electromagnet consists of 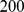 winds of 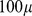
m thick enamelled copper wire wound around a low carbon steel core of 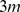
m diameter and 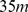
m length which was tapered to 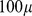
m towards the sample. The electromagnet was connected to a regulated DC power supply (model LC3210; Aplab, India) capable of supplying 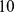 Amperes of current through a toggle switch. The magnet was covered in Teflon tape to prevent contamination of the sample solution and to prevent the accumulation of debris on the electromagnet tip. The magnet was mounted on a three axis stage (Newport, USA) with motorised actuators (Newport, USA) which can be controlled through the computer by RS232 commands to the controller (Newport, USA).

### Force protocol and Imaging

**Figure 1:**
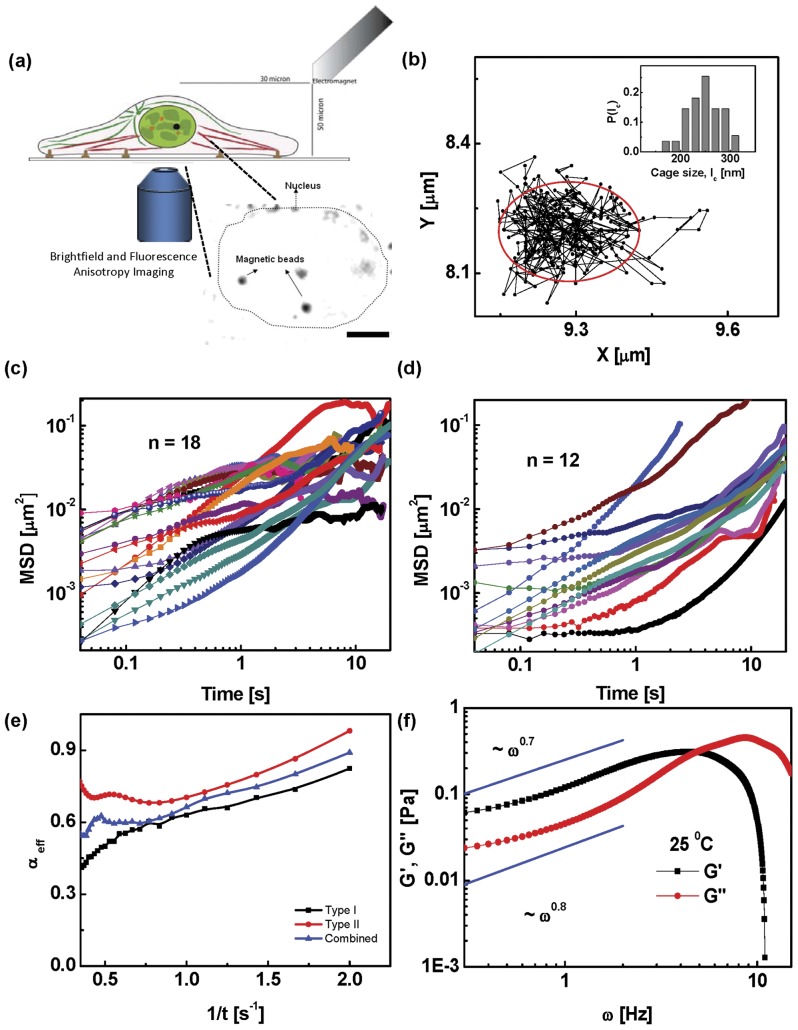
Single particle tracking analysis of particles in the nucleus. (a) Schematic of the experimental setup. An electromagnet mounted on a fluorescence microscope is used to apply forces on a 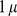
m paramagnetic bead microinjected into the nucleus. (Inset) Brightfield image of the nucleus showing microinjected magnetic beads. The dotted line represents the edge of the nucleus. Scale bar 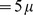
m. (b) Typical trajectory of a bead in the nucleus of a living cell at 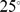
C showing caging behaviour. (Inset) Histogram of cage sizes 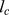 across 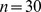 beads shows a narrow distribution about 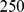 nm. (c & d) Mean square displacement versus time for beads in the nuclei of untreated cells in the absence of force. 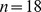 beads show Type I behaviour and 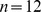 beads show Type II. (e) Mean effective exponent 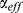 versus inverse time for Type I and II trajectories together with the combined data. (f) Shear and loss modulus, 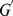, 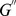 (averaged over all beads) as a function of frequency 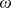. At low 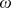, the response of the nucleus is elastic (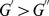) and crosses over to a viscous response at higher 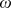. Fits at low 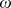 show that the nucleus behaves as a power-law solid at low frequencies.

The coverslip dishes were mounted on the microscope and the tapered tip of the electromagnet was moved to a position 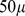
m vertically and 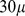
m horizontally distant from the bead in the nucleus (Fig. 1). The cells were then imaged in fluorescence mode at 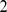 s binning time for anisotropy of H2B-EGFP and then in brightfield at 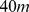
s bin time for 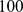 s to track the bead. The electromagnet was then switched on at a current of 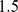 A to provide a force of 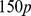
N at the bead. The brightfield imaging was repeated followed by the anisotropy imaging. The images were then processed using custom programming ImageJ and LabVIEW. The beads were tracked till the point they were lost due to vertical movement. The trajectory was plotted, and the mean square deviation (MSD) and the diffusion parameter 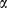 were computed. The caging sizes of the diffusing partciles were calculated by plotting a 2D spatial histogram of occupancy of the bead centroid. All points of bead position were used to calculate the frequency of bead presence in each 40 nm×40 nm area, followed by visual identification of the peaks and 1D fits along X and Y axes passing through the peak to calculate of the full width at half maximum of the occupancy peaks. The experiment was carried out at 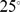
C in phosphate buffered saline (PBS; 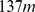
M NaCl, 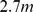
M KCl, 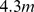
M NaH
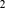
PO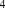, 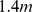
M K
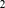
HPO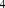, pH - 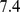). All image analysis, data analysis, and data plotting were conducted using ImageJ, LabVIEW and Origin respectively.

### Microinjection


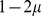
m paramagnetic polystyrene beads (Polysciences Inc., USA) were diluted 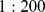 in MilliQ water and loaded onto custom pulled glass micropipettes with a pore size 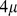
m. The cells were visualised using and optical microscope and the beads were microinjected into the nuclei of the cells using a Femtojet microinjection setup (Eppendorf Inc, Germany). The microinjection parameters were adjusted to get 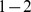 beads per nucleus with a minimum amount of injection volume. The cells were then incubated in medium or the appropriate buffer at 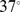
C for 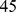 minutes for them recuperate from the shock of microinjection. They were then mounted on the microscope for the experiment. For the labelling of transcription compartments, the cells were microinjected with 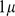
M Alexa
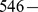
UTP in the cytoplasm immediately after bead microinjection. The cells were then incubated in medium at 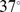
C for 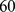 minutes prior to the experiment.

### Fluorescence Anisotropy imaging of chromatin assembly

A custom made module was used to acquire images for fluorescence anisotropy measurements. A linearly polarised excitation beam obtained by passing the Arc Lamp beam through a sheet polariser (New Focus, USA) and excitation filter (HQ480/40×; Chroma Tech, USA) was used to excite the cells and the emission was split into parallel and perpendicular polarisations using a Polarizing Beam Splitter (New Focus, USA) after passing it through an emission filter (HQ535/50 m; Chroma Tech, USA). The parallel and perpendicular images were acquired on two halves of an Andor iXon 887BV EMCCD camera. The images were acquired at 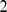 s exposure for each image. Anisotropy was calculated from the split image on a pixel by pixel basis after smoothing the original image. For each pixel, the anisotropy was calculated using the formula, 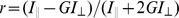 where, 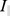 is the intensity recorded on the pixel in the parallel image, 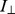 is the intensity recorded on the corresponding pixel in the perpendicular image, and 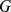 is a factor to compensate for the difference in sensitivity of the detection system for parallel and perpendicular components of the light.

## Results

### Caging of large passive particles: active solid

We first study the dynamics of paramagnetic beads of diameter 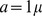
m, microinjected into the nucleus of HeLa cells stably transfected with H2B-EGFP [2]. Each microinjected nuclei contains only 1 bead, nuclei with more than one bead are typically unhealthy showing blebbing and are washed away on replacing medium. Thus a two-point microrheology experiment, though desirable, is practically unfeasible for beads of this size. Even in cells with only one bead in the nucleus, 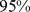 of the cells undergo cell death within 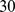
min and are removed on washing after the incubation. The cells which are viable and stuck to the coverglass showing no signs of blebbing after the 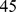
min recuperation period are used for the experiment. The position of the beads was tracked using brightfield imaging at 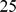
Hz for 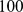
s (Fig. 1a). Experiments were done at both 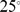
C and physiological temperatures (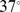
C). The beads move predominantly as a result of active stress fluctuations in the embedding nuclear medium (as seen by comparing the dynamics under conditions of ATP-depletion). By analyzing the diffusion of micron size beads, we probe the microrheology of the fluctuating nuclear medium on scales of order 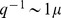
m, the size of the bead.

From the time series of the displacement of the bead, 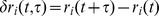 where the subscript 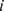 labels the 2d vector index (projection along the x-y plane), we can compute the mean square displacement (MSD), 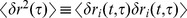 (repeated indices 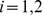 are summed over. We have also checked that the motion in the x-y plane is isotropic.). We find that at both temperatures, the beads do not move much and appear caged (Fig. 1b). The bead trajectories show two distinct behaviours, which we label Types I and II (Fig. 1c–d), which arise due to heterogeneity in the nuclear medium. Most trajectories (Type I, 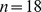) show a plateauing of the MSD at longer time scales, the remaining (Type II, 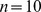) show a monotonically increasing MSD over a 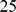
s time scale (beyond this time, the data is very noisy). We have discarded those trajectories where the beads went out of focus within 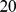
s. Rather than fitting the entire MSD curve to 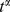, we compute a local effective 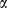, given by 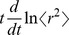, which we plot against 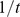 (Fig. 1e). Both Type I and II trajectories show that 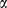 decreases with increasing time, indeed the decrease in 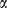 is more significant for the more numerous Type I trajectories (and hence the combined data set).

We interpret the low value of the asymptotic 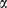 as implying that the bead is caged within a small region, the cage diameter is sharply distributed about 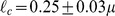
m (Fig. 1b(inset)). This caging scale increases slightly at the larger temperature. Note that our measurements cannot reveal whether the small movement of the bead is due to advection by the nuclear mesh or movement relative to a stationary mesh, and therefore we might expect the effective value of 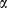 to be even lower.

We use the fourier transform of the MSD of the bead, 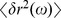, to probe the microrheology of the nucleus at the scale of a micron. For equilibrium viscoelastic fluids, the measured 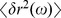 together with the generalized Stokes-Einstein relation (GSER) [17], [18], 
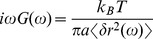
, can be used to determine the frequency dependent shear and loss modulus, 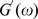 and 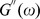, respectively, where 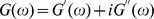 (we will henceforth take 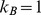). However, the nuclear medium is an active medium, subject to active systematic and fluctuating stresses arising from the chromatin remodeling machines bound to the nuclear meshwork. In an *active* context, 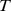 in the GSER is replaced by 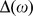, the power spectrum of the active stress fluctuations [19]. Modeling the active stress fluctuations as arising from a isotropic suspension of active force dipoles of concentration 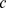 and strength 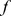 embedded in the nuclear medium of viscosity 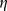, one can obtain 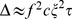 at low frequencies, where 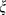 is the mesh size and 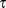 represents the characteristic time scale over which the active stresses are correlated [20], [21]. Since 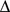 is dimensionally related to an activity temperature multiplied by a viscosity, 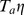, this provides an estimate of the active temperature [20], [21].

Analyzing both the Type I and II MSD data, we find that at the lowest frequency (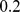
Hz), 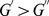, i.e., the response is largely elastic, suggesting that the nucleus behaves like an isotropic, active solid at the scale of the bead (Fig. 1f). We note that the typical magnitude of 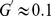
Pa at the lowest frequency, which is one order of magnitude smaller than the average obtained from corresponding microrheology studies in fibroblasts [22], but falls within the range observed in their experiments. This difference could possibly be attributed to the variation in nuclear architecture of the two cell types. From a fit to the data at low frequencies (Fig. 1f), we find that 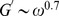, while 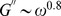, the nucleus thus behaves as a power-law solid at low frequencies, 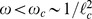. At high frequencies (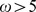
Hz) there is a crossover to a viscous response, 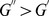. These qualitative results are replicated at 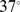.

Taken together, our data suggests that the micron size passive beads are caged by the nuclear meshwork, which may be characterized by a confining (caging) potential. Our results suggest that the nucleus behaves as an active amorphous solid when probed at scales larger than the mesh size.

### ATP dependent yielding of passive particles

We now analyze the movements of the bead when subject to an applied force. We use a gradient magnetic field to apply forces on the paramagnetic beads, giving rise to both shear and stretching deformations of the nuclear meshwork (Fig. 1a). For low values of the applied force (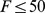
pN), we see no qualitative change in behaviour of the passive particles – the beads are still confined by the meshwork, although the spatial extent of confinement appears a little larger, owing perhaps to the fact that the meshwork is deformed affinely.

**Figure 2:**
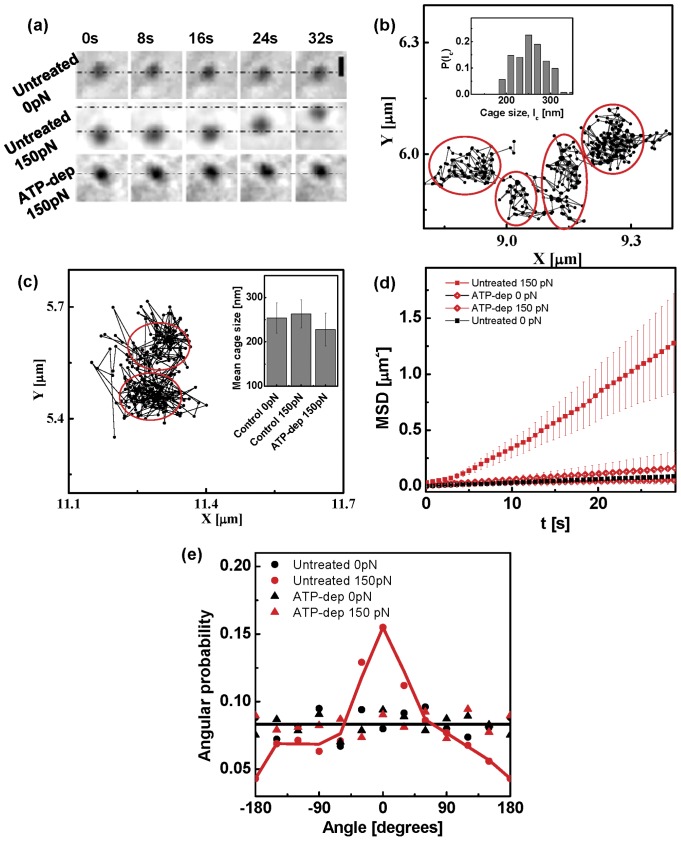
Force induced displacement of particles in the nucleus. (a) Time sequence of brightfield images of typical beads in the nuclei of untreated cells in the absence of force and under a super-threshold 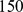 pN force, and in ATP depleted cells under 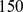 pN force, respectively. The dotted lines are guides to the eye. Scale bar 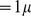
m. (b) Typical trajectory of a bead in the nucleus of a living cell at 25

Beyond a threshold force 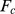, which lies between 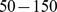 pN, the motion qualitatively changes – the beads become unconfined (some move as far as 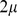
m, a fair fraction of the nuclear size 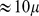
m diameter) and the material yields (Fig. 2a). Such yield forces are not high enough to rip the meshwork [23], i.e., neither does the filamentous meshwork break, nor do the crosslinkers that hold the mesh together, unbind. We observe that under such conditions, the beads exhibit extended trajectories (Fig. S1), which on closer inspection, display hops between confined patches, whose size is comparable to the caging scale 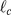 (Fig. 2b).

C under a 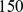 pN force shows cage-hopping dynamics with a distribution of cage sizes given in Inset. (c) Typical trajectory of a bead in the nucleus of a living cell depleted of ATP at 25

C under a 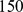 pN force shows caging behaviour with hardly any hopping. (Inset) Mean cage size under ATP-depleted and control conditions. While the mean cage sizes are roughly similar, the untreated cell shows cage-hopping dynamics in the presence of a threshold force. (d) Mean square displacement averaged over many beads (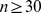) versus time in the nuclei of untreated cells and ATP depleted cells under 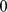 pN force and 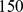 pN force. (e) Probability distribution of the angle between the instantaneous displacement vector of the bead and the direction of the external force, shows that for untreated cells under a 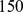 pN force, the beads move predominantly in the direction of the force. In all other cases the bead movement is isotropic.

In contrast, the bead movement is completely stalled when the same experiment is performed on cells depleted of ATP (Fig. S1). In the absence of an applied force, the bead movements of the ATP-depleted cells are comparable (though slightly smaller) to the untreated cells, suggesting that that the caging scale 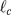 is similar (though slightly smaller in the ATP-depleted case). In the presence of a force of 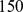
pN, there is still very little movement in the ATP-depleted cells (Fig. S1). A closer look shows that the trajectories either show confined diffusion in one patch or at most two patches; the caging scale is roughly the same, 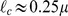
m (Fig. 2c). This indicates that the inter-cage hops observed in the untreated cells is an activated process, and suggests that the mechanism for yielding is an ATP-dependent mechanism. In a later section, we will provide evidence that this ATP-dependent mechanism involves the dynamic remodeling of chromatin fibers.

The MSD computed from the trajectories in the presence of the 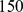 pN force, shows extended movement in untreated cells and negligible movement (caging) under conditions of ATP-depletion (Fig. 2d). The angular distribution of the bead displacement vectors with respect to the direction of the force, shows that the beads move predominantly in the direction of the force (Fig. 2e).

This cage-hopping in the presence of an applied threshold force is observed at both 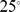 and 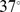
C, and can be described as an escape over a barrier, 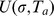, which depends on both the local stress 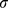 and the active temperature 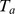. The local stress is a sum of the local active stress and applied stress. Cage escape occurs when 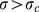, the threshold yield stress.

In the next section we show that the internal force generators that strain the nuclear meshwork non-affinely and facilitate bead movement, are ATP-dependent chromatin remodeling agents.

### Chromatin remodeling facilitates particle movement

To decipher the molecular processes behind the activity dependent un-caging mechanism of large beads, we perform the microrheology experiments on cells in which specific aspects of the chromatin mesh work have been perturbed. We study the bead movement in cells which were treated to have (a) condensed chromatin, (b) decondensed chromatin, and (c) in cells which were RNAi depleted of the nuclear scaffold protein lamin B1. Chromatin decondensation was achieved using Trichostatin A (TSA), an inhibitor of Histone deacetylases [2], [24]. The condensation of the chromatin architecture was accomplished by causing apoptosis using the protein kinase inhibitor Staurosporine [2], [25]. The depletion of lamin B1 leads to a detachment of the physical coupling between the cytoskeleton and the chromatin architecture, an area that has been extensively studied in recent years [26], [27], [28], [29]. The lamin and chromatin mesh organization is affected with the nuclei showing a decreased volume with higher levels of euchromatin in the lamin free regions [16], [30]. Further Lamin B1 silencing also alters gene expression and changes Pol II activity while localising TCs [2], [30], [31].

**Figure 3:**
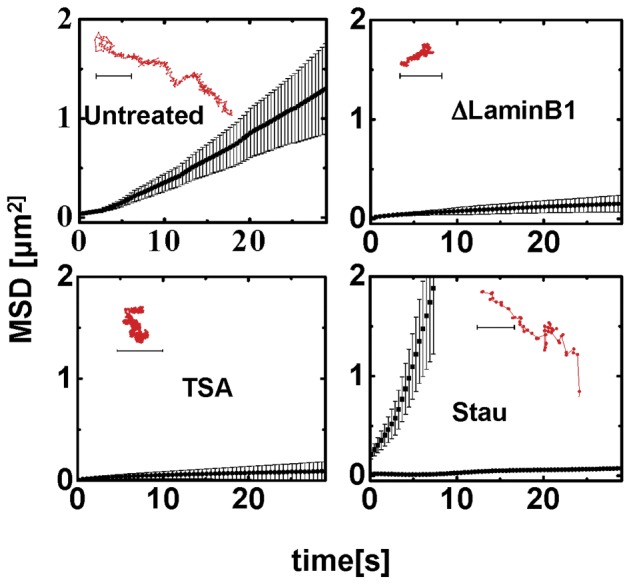
Functional nuclear perturbations alter particle displacement. Mean square displacements of the beads in the nuclei of cells under 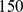 pN force at 25

All the chromatin perturbations led to a dynamical arrest of the beads even in the presence of the applied force (Fig. S1, Fig. 3), though the results of the Staurosporine treatment were a bit ambiguous, possibly because being a global kinase inhibitor, Staurosporine can in principle (de)activate several signaling pathways. Dynamical arrest upon chromatin perturbations is reflected in the smaller values of the mean cage size (Fig. S2b), and the values of mean 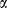 extracted from fits to the MSD. Taken collectively, these results suggest that the molecular processes responsible for the cage-hopping of the bead beyond a yield force are active stress fluctuations arising from ATP-dependent chromatin remodeling proteins.

C, upon treatment with chemical perturbations of chromatin remodeling, as indicated (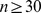). (Insets) Trajectories of typical beads in the nuclei of cells under 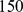 pN force on treatment with chemical perturbations of chromatin remodeling.

### Active porosity as a structural basis for yielding

To understand how the active force generators might deform the chromatin meshwork, we study the spatial organization of chromatin at high resolution, with and without the external force. The subtle differences in rotational diffusion of the EGFP tagged to H2B have earlier been used to probe the extent of chromatin packing within the nucleus [2]. Here we utilized this technique to understand the mechanism of the force-induced movement of the bead. The pulled bead exerts forces on the chromatin and alters the local chromatin architecture, which we monitor by following the local fluorescence anisotropy of labeled H2B.

From a rheological perspective, locally, nuclear chromatin exists in two states – it is either tightly bound in the histone complex higher order structure or is relatively unbound – let us define the local volume fraction of the bound chromatin as *chromatin compaction*. In principle, there are a multitude of intermediate states between the tightly bound and unbound, but for the present purposes this simplification suffices. Chromatin compaction may be conveniently measured by measuring the local volume fraction of bound (unbound) histones, which in turn can be measured by their constrained (unconstrained) rotational diffusion. By labeling H2B (a member of the histone complex) with GFP, we can locally measure the steady state fluorescence anisotropy of H2B-EGFP, which by the Perrin formula [32] is a direct measure of the rotational diffusion of the labelled histone H2B. Higher values of anisotropy correspond constrained rotational mobility of the fluorophore and hence to higher compaction of the chromatin. The local anisotropy is a weighted sum of the anisotropies from the different states. In this way, the spatial map of the fluorescence anisotropy of H2B-EGFP is a measure of the spatial distribution of chromatin compaction within the nucleus [2]. Note that for this interpretation to hold, we have to ensure that the fluorescence anisotropy is not a result of energy transfer between proximal fluorophores (homoFRET), which we do by making the levels of labeled H2B low, around 5% of the native H2B, so that the typical distance between fluorophores is too large for energy transfer to be appreciable. We verify this by explicitly demonstrating that at these levels of labeling, the anisotropy does not change upon photobleaching [2]. In addition, we show the consistency of this interpretation, by demonstrating that the spatial map of the anisotropy gives rise to the same banding pattern of polytene chromosome condensation of Drosophila as is obtained using more traditional methods [2].

**Figure 4:**
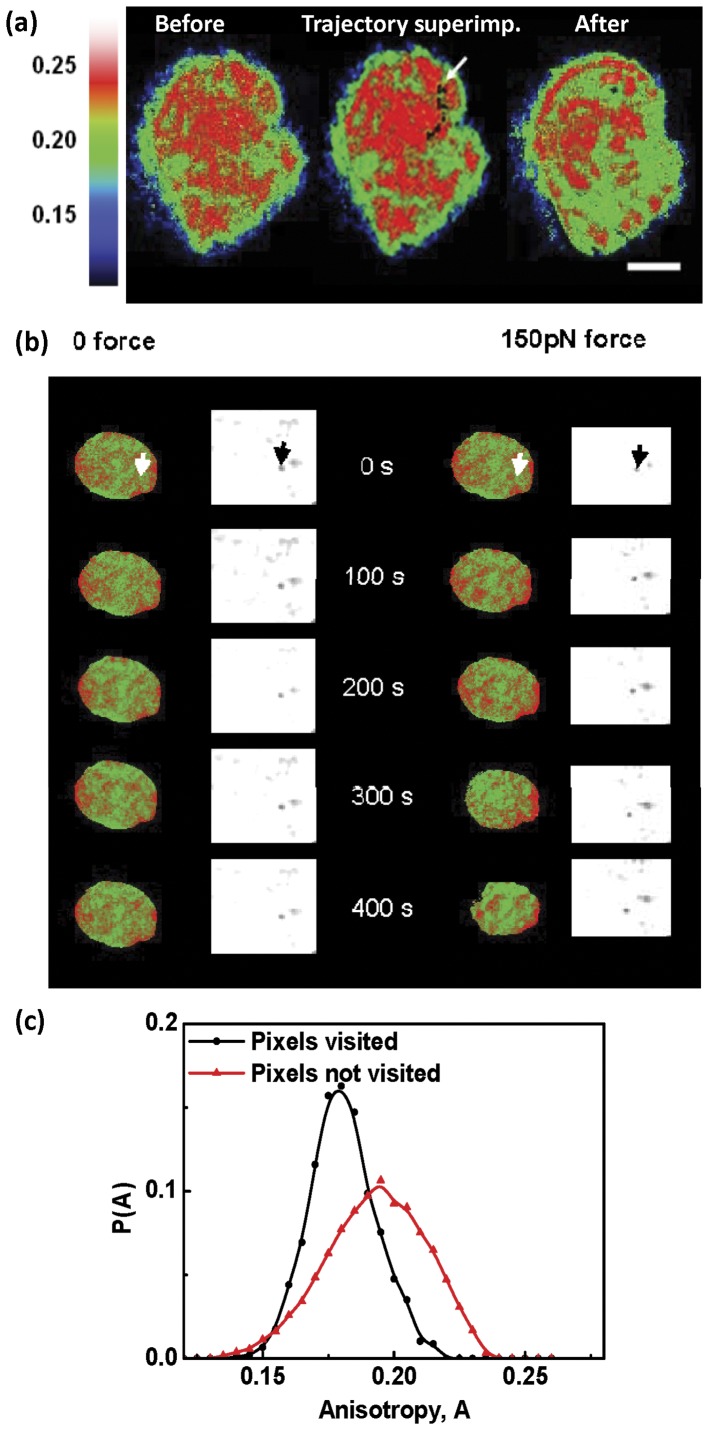
Visualising chromatin remodeling during particle displacement. (a) Spatial map of fluorescence anisotropy of the chromatin (H2B-EGFP) assembly before and after movement of the bead through the nucleus. The black dots represent the trajectory of the bead and the start point is marked by the white arrow. Scale bar 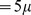
m. (b) Time course of anisotropy maps of chromatin on application of force on a 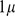
m paramagnetic bead in the nucleus. Arrows indicate the initial position of the bead. (c) Histogram of anisotropy of pixels visited by the beads in the nuclei (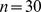) and anisotropy of pixels not visited by the beads as extracted from anisotropy image acquired before bead movement.

Here we imaged the fluorescence anisotropy of the H2B-EGFP using a split anisotropy imaging on single camera chip arrangement, with a spatial resolution of 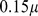
m. The fluorescence images acquired before and after the movement of the beads in the absence of a force, suggested that spatial distribution of chromatin compaction is unaltered by the force-free movement of the beads (Fig. S3b, Fig. 4b), consistent with it being passive.

The volume fraction of the bound histones is clearly set by the energy barrier between the bound and unbound states of the histone. Application of an external force strains the chromatin mesh and reduces this energy barrier by an amount linear in the force (or 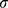). This should increase the fraction of unbound histones, leading to lower chromatin compaction. The anisotropy maps of the untreated cells, acquired before and after the application of force, showed a decrease in the mean anisotropy of the nuclei after the movement of the beads (Fig. S3a), consistent with a reduced mean chromatin compaction. Indeed while the force is turned on, the mean anisotropy systematically decreases (Fig. S3b). When we overlay the bead trajectory on the anisotropy maps, we find that the bead trajectories typically lie along regions of low anisotropy (Fig. 4). In Fig. S3c, we show that the pixels occupied by the trajectories of the beads are strongly correlated with those where the anisotropy is low (lower chromatin compaction).

The strong correlation between the regions of low chromatin compaction and the bead movement, provides a new insight into the physical state of nuclear matter during yielding. Our results suggest that the yield behaviour is associated with the actively generated free-volume (porosity) in the nuclear mesh arising from chromatin remodeling machines.

### Phenomenological Model

Here we take a phenomenological approach to the caging dynamics of a tagged bead embedded in an active isotropic nuclear gel with a finite yield stress. We represent the caging of the bead by a caging potential with a barrier height which depends on the active temperature, 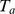. In the absence of a force, this barrier height is high, and so the escape rate from the cage is exponentially suppressed, 
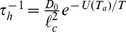
 (here, 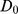 is the tagged-particle diffusion coefficient within the cage of size 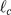 and 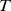 is the solvent temperature). Following Eyring [33], we propose that the barrier height of the caging potential reduces in the presence of an applied stress by
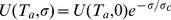
(1)which implies that beyond a yield stress 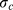, the bead can hop out of the confining potential. The escaped bead then moves ballistically (in the large force regime) till it gets captured by another cage, and hops out again; the direction of the motion is correlated with the direction of the applied force (Fig. 2e). Thus the mobility is given by 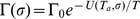. This form of the stress-dependent caging potential is the simplest realization of activated hopping and incorporates the linear dependence on stress (for small 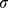) assumed in Eyring's work. Alternatively, yielding could arise from a strain criterion, when the local free volume (local mesh size) altered by the applied stress, exceeds the size of the bead 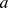 [34]. This active extension of the Eyring model [33] is in qualitative agreement with our observations for the microrheology of the passive bead.

### Transcription compartments are active particles at 37

C

How do intrinsically embedded large nuclear particles, such as transcription compartments, move in the nuclear medium?

To address this, we enquire about the nature of the force-free (and dependent) movements of TCs at both 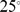 and 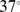
C.

**Figure 5:**
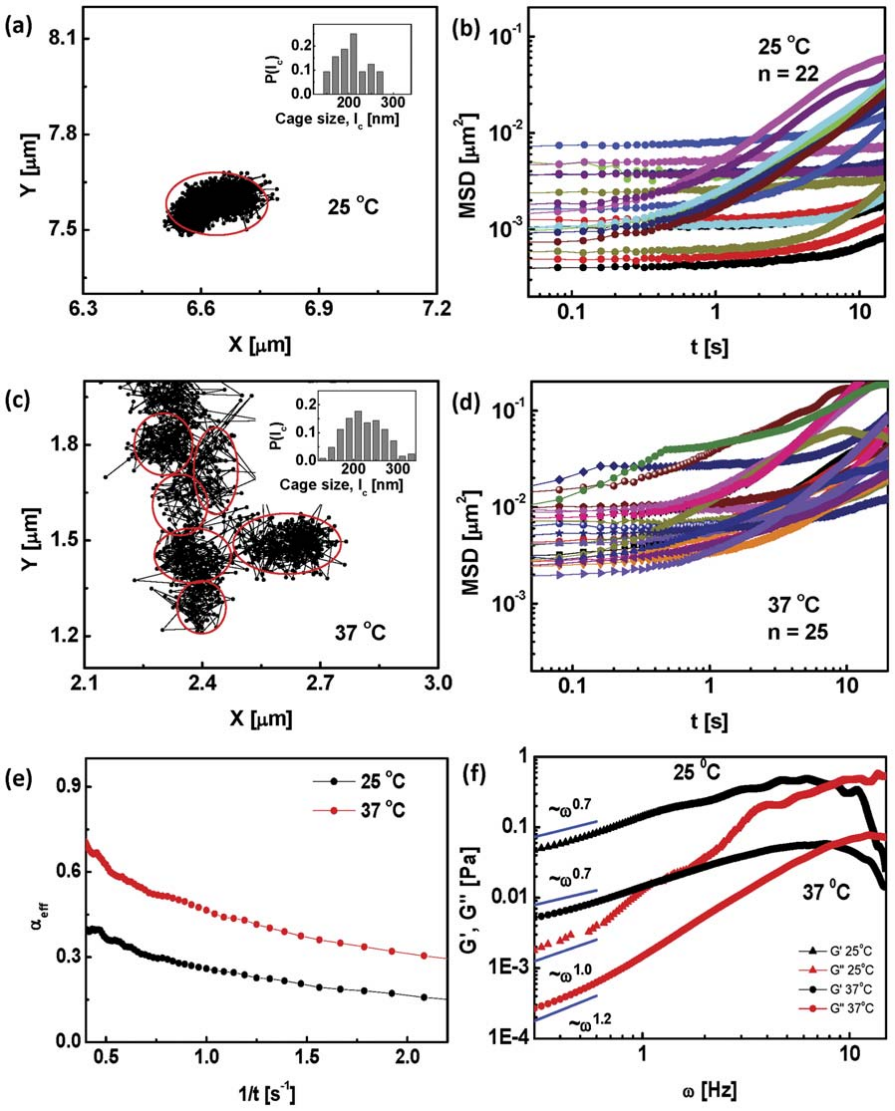
Dynamics of active particles in the nucleus. (a) Trajectory of TF in the nucleus of a living cell at 25

Recognizing that the TCs are highly concentrated in UTP, our strategy was to image the TCs using fluorescently labeled UTP. We therefore serially microinjected the cells with the probe bead (for force manipulation) and Alexa546-UTP, the latter forms distinct foci inside the nucleus. We tracked the position of the UTP-foci using fluorescence imaging, both under conditions where there is no force on the bead and when the bead is subject to a step force of magnitude 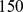
pN. In the force-free experiment, we tracked the position of the TCs using fluorescence imaging at 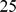
Hz for 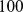
s (Fig. 5).

C shows caging. (Inset) Probability distribution of cage sizes is similar to that of the beads. (b) Mean square displacement of the TFs in the nuclei (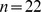) in the absence of force at 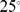
C. (c) Trajectory of TF in the nucleus of a living cell at 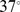
C showing dramatic cage-hopping dynamics over long distances even in the absence of an external force. Inset shows the distribution of cage sizes. (d) Mean square displacement of TF in the nuclei in the absence of any force at 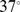
C. The effective 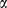 exponent obtained by fitting is reported in the main text. (e) Consistent with the above, the low frequency (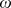) behaviour of the mean 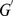, 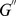 of TFs shows an elastic response, at 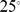
C and at 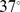
C. The figure shows the power law fits to the data, which in the absence of a quantitative active rheology model should merely be taken as indicative.

At 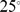
C, as observed in [5], [31], we find that the TC's show the same caged movement as the 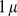
m beads (Fig. 5a), with cage sizes comparable to the bead (albeit with a slightly broader distribution than the bead). In a few instances, there was some amount of inter-cage hopping, but not a whole lot. The MSD of these trajectories again shows two populations, one of which shows almost immobile behaviour over 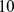
s (Fig. 5b). The effective 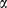 exponent has a mean value 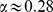 (comparable to the value of 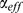 for the beads). A microrheology analysis done on these trajectories shows that at the lowest frequency (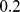
Hz), 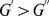 (Fig. 5e).

On the other hand, as observed in [5], [31], the TCs at physiological temperatures, 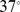
C, display significant movement *even in the absence of any applied force* (Fig. 5d). The force-free TC trajectories at 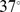
C show a cage-hopping behaviour akin to the trajectories of the bead in the presence of a force (Fig. 5c). The mean cage size is around 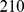
nm and the TCs move through regions of low chromatin compaction (data not shown). This large scale movement of TCs was shown to be similarly affected by ATP depletion and perturbations to chromatin remodeling [5], [31]. Also, the movement characteristics of the chromatin itself has been shown to be affected by ATP depletion [35]. Analysis of the 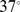
C MSD data (Fig. 5e) showed that the mean 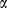 is significantly larger (around 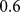) compared to its value at 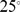
C. Microrheology analysis on the MSD at 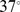
C still shows that at low frequencies, 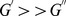 (Fig. 5f), however the elastic moduli is an order of magnitude lower than its value at 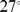
C.

**Figure 6:**
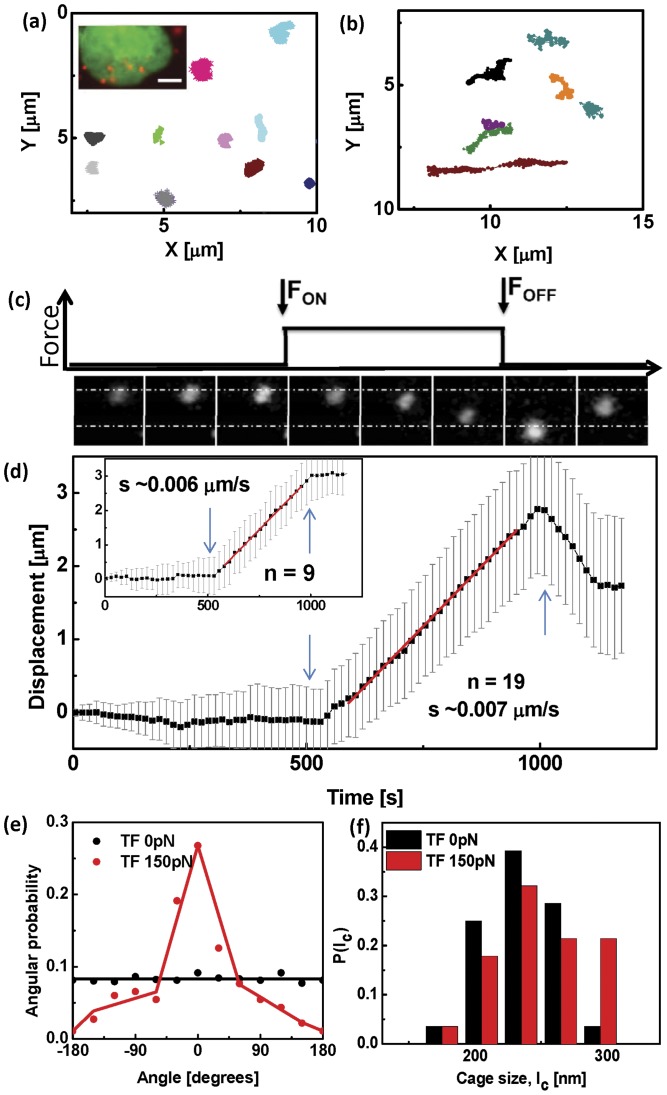
Force transduction within the nucleus. Typical trajectories of TFs in the nuclei of cells at at 25

We next apply a step force of 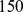
pN for 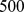
s at 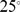
C, this time scale is longer than the total observation time employed to study the force-free movement of the beads. We collected data for those situations where the bead was some distance away (typically, 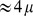
m) from the targeted TCs. Just as in the case of the passive beads, the UTP-foci show large scale directed movement when a threshold force is applied on the bead at 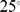
C (Fig. 6a–b).

C, (a) in the absence of force and (b) under a 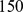 pN force, applied on beads. (Inset) Fluorescence image showing H2B-EGFP marked nucleus in green and alexa546-UTP foci in red. Scale bar 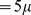
m. (c) Fluorescence images of the TFs at every 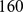 s showing directed movement of the compartments in the presence of force. The black line represents the force profile. (d) Type I and Type II trajectories of Alexa546-UTP labelled TCs with 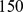 pN force applied on 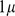
m paramagnetic bead for 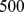 s. TFs in the nucleus show directed movement in the same direction as the bead. Error bars denote standard deviation. (e) Probability distribution of the angle between the instantaneous displacement vector of the TFs and the direction of the 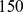 pN external force, shows that the TFs move predominantly along the direction of the force. In the absence of a force, the movement of the TFs is isotropic.

The response to the step force shows interesting features. After the initial linear increase of the displacement of the TCs with time, we find that in most cases (Fig. 6c, 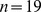) there is a sharp drop as soon as the force is turned off, followed by a plateauing out to a residual strain. The typical values of the residual strain are around 0.6 times maximum strain, with some cell-to-cell variation. This residual strain is reminiscent of creep in *glassy* materials, here generated by the active remodeling of the nucleoskeletal meshwork as it is deformed by the force on the magnetic bead. In the rest of the cases, (Fig. 6c(inset), 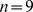), the strain does not show a drop but continues increasing even when the external force is released. This ‘inertia-like’ behaviour is again a consequence of the active remodeling of the nuclear mesh. In either case, the TC's move predominantly in the direction of the applied force (Fig. 6e).

These results taken together suggest that at 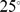
C, the TC's behave roughly like the large passive beads. However at 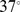
C, the TC's behave as active particles, which by coupling to the chromatin meshwork and remodeling agents, can act upon the mesh (in addition to being acted upon by the active stress fluctuations of the nuclear mesh). Such active particles would locally remodel the nuclear meshwork, even reducing the yield stress 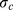 to zero.

## Discussion

It is evident that the spatiotemporal organization of transcription reactions (and hence information) must be *actively* regulated and maintained by the nucleus. To achieve this large nuclear bodies such as transcription compartments need to be targeted to specific gene loci at precise times. This requirement makes two apparently opposing demands on the physical environment of the nucleus – the nuclear medium must be *rigid* enough to maintain the 3D positioning of the gene loci, on the other hand it must be *fluid* enough to allow for *specific* transcription agents to be driven towards their gene targets at *specific* times.

In addressing this, we were motivated by our recent observations on transcription compartments [5] which revealed large scale movement at physiological temperature (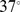
C) but hindered movement when ATP was depleted or TSA added, suggesting a link between chromatin assembly and nuclear body movements. We therefore probed the movement of micron-size passive beads inserted into the nucleus at two different temperatures (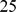 and 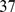 
C), both in the absence and presence of an external force. Since the moving beads have to make their way through the dense nuclear medium, we simultaneously looked at the distortion in the local chromatin meshwork using fluorescence anisotropy.

Our results show that the micron-size beads are caged by the nuclear meshwork at both temperatures. Similar studies using magnetic nanorods exhibit the same caging behaviour [36]. On applying a threshold force of around 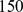
pN, the beads move by cage-hopping. This movement is driven by active stresses generated by ATP-dependent chromatin remodeling proteins, which actively generate free-volume (porosity) in the nuclear mesh. At a rheology level, our results suggest that the nucleus behaves like an active amorphous solid with a finite yield stress when probed at the scale of a micron. The solid component provides the rigidity while the actively generated porosity provides the fluidity, thus meeting the two opposing demands alluded to above.

A similar study done on the movement of labeled transcription compartments at 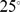
C, both in the presence and absence of a force, shows its behaviour is identical to the micron-scale beads at 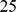 and 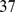 
C – they behave as passive particles moving through an active medium [15], [37]. This is in contrast with the movement of TCs at 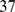 
C [5], where they exhibit large scale movement *even in the absence of an external force*. This movement is ATP-dependent and is driven by the active stresses generated by chromatin remodeling proteins. The similarlity between the force-free movement of TC's at 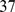 
C and the movement of TC's at 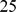 
C in the presence of a threshold applied force, suggests that at physiological temperatures, TCs apply *active internal forces* which locally remodel the chromatin meshwork, facilitating movement towards gene loci. In the nomenclature of [15] they are active particles moving in an active medium.

Our work suggests that the active remodeling of the nuclear chromatin meshwork via stresses generated by ATP-dependent chromatin remodeling proteins may also contribute to the spatiotemporal regulation of transcription reactions in the living cell nucleus. The active remodeling of the chromatin meshwork may drive particle currents and hence target nuclear bodies to specific gene loci. We have discussed such mechanisms in a recent theoretical paper [37] and plan to explore these issues experimentally in future.

## Supporting Information

Figure S1
**Typical trajectories of beads in the absence of force and under** 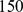 **pN force in control cells and cells subject to different perturbations at** 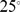 **C.** Scale bar is 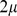
m.(TIF)

Figure S2
**Caged movement of particles inside the nucleus.** (a) Mean cage sizes of the beads in untreated cells and under perturbed conditions and TFs in untreated cells at 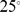
C. (b) Mean values of the movement of the paramagnetic bead inside the nuclei of cells under different perturbations. * is 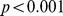. Error bars denote standard deviation.(TIF)

Figure S3
**Chromatin packaging mapped as anisotropy of H2B-EGFP.** (a) Mean anisotropy values of H2B-EGFP in control and in treated cells before and after application of force. * denotes 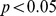. (b) Variation of mean anisotropy with time shows that in the absence of force, the anisotropy is roughly a constant, while it decreases and saturates under the application of a 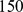 pN force. Error bars denote standard deviation.(TIF)
