# Supplementary figures and images for: Dynamics of Passive and Active Particles in the Cell Nucleus
Source: PLoS One. 2012 Oct 15;7(10):e45843. doi: 10.1371/journal.pone.0045843 (PMC3471959; doi:10.1371/journal.pone.0045843)

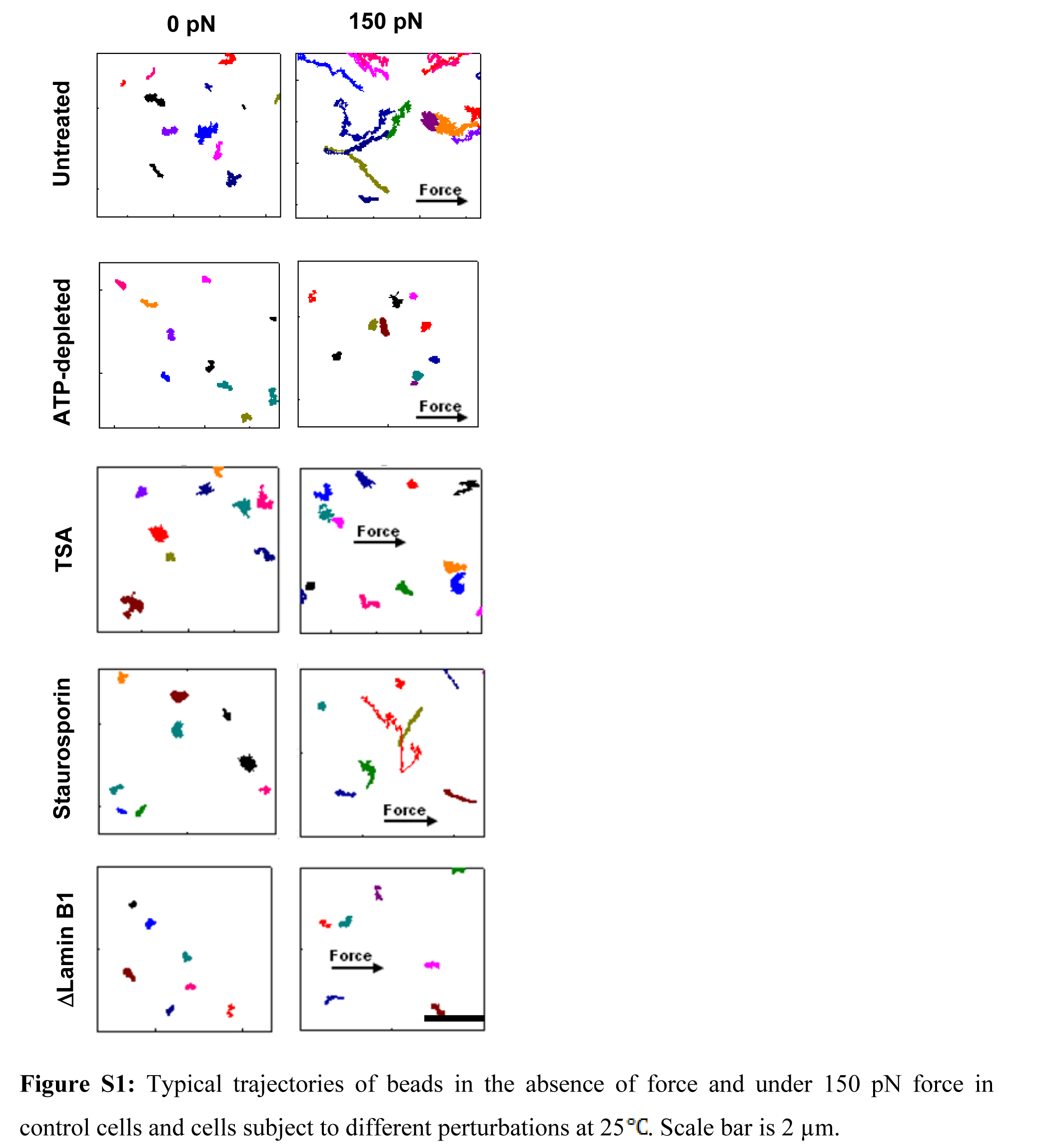

Supplement: Figure S1 — Typical trajectories of beads in the absence of force and under pN force in control cells and cells subject to different perturbations at C. Scale bar is m. (TIF) [file pone.0045843.s001.tif]

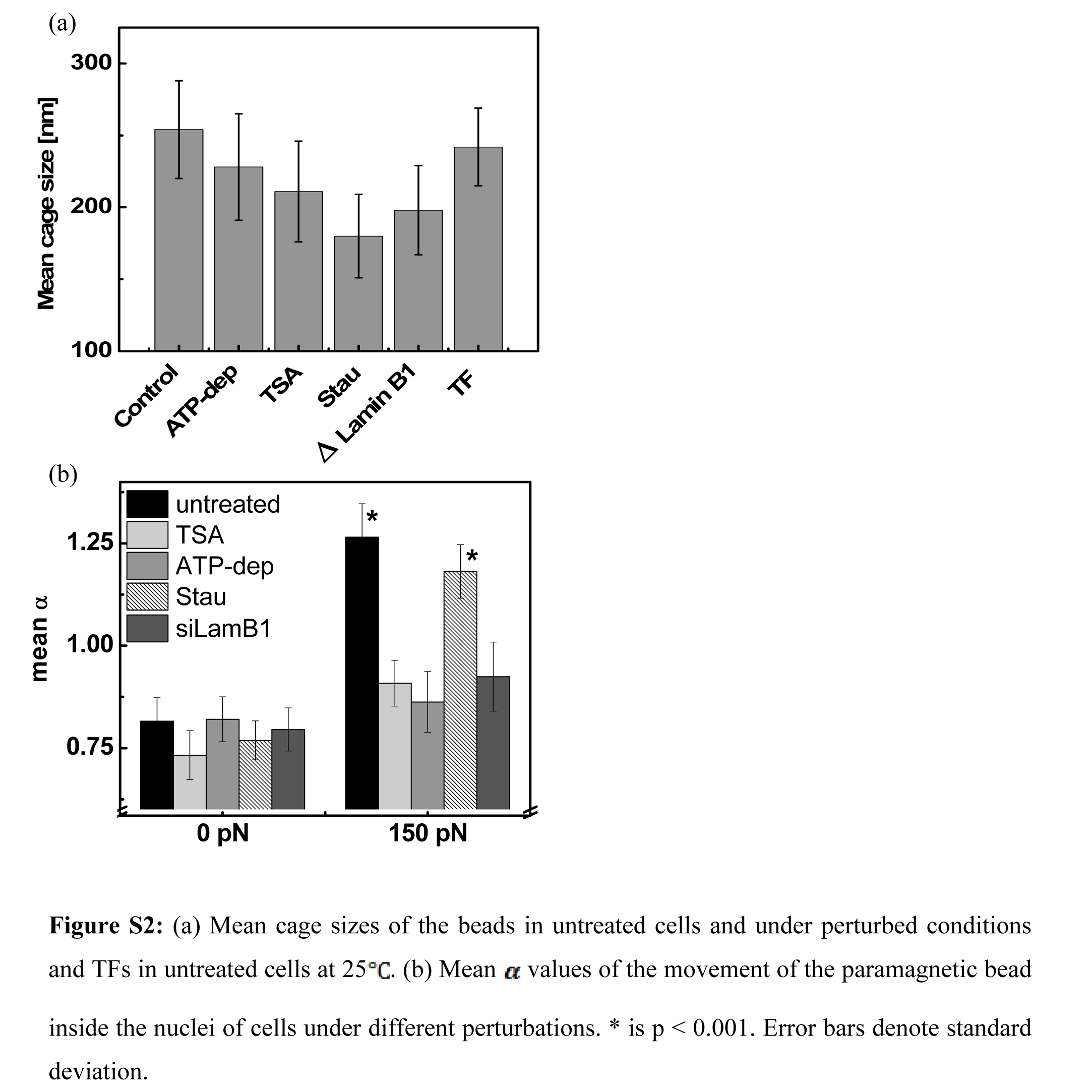

Supplement: Figure S2 — Caged movement of particles inside the nucleus. (a) Mean cage sizes of the beads in untreated cells and under perturbed conditions and TFs in untreated cells at C. (b) Mean values of the movement of the paramagnetic bead inside the nuclei of cells under different perturbations. * is . Error bars denote standard deviation. (TIF) [file pone.0045843.s002.tif]

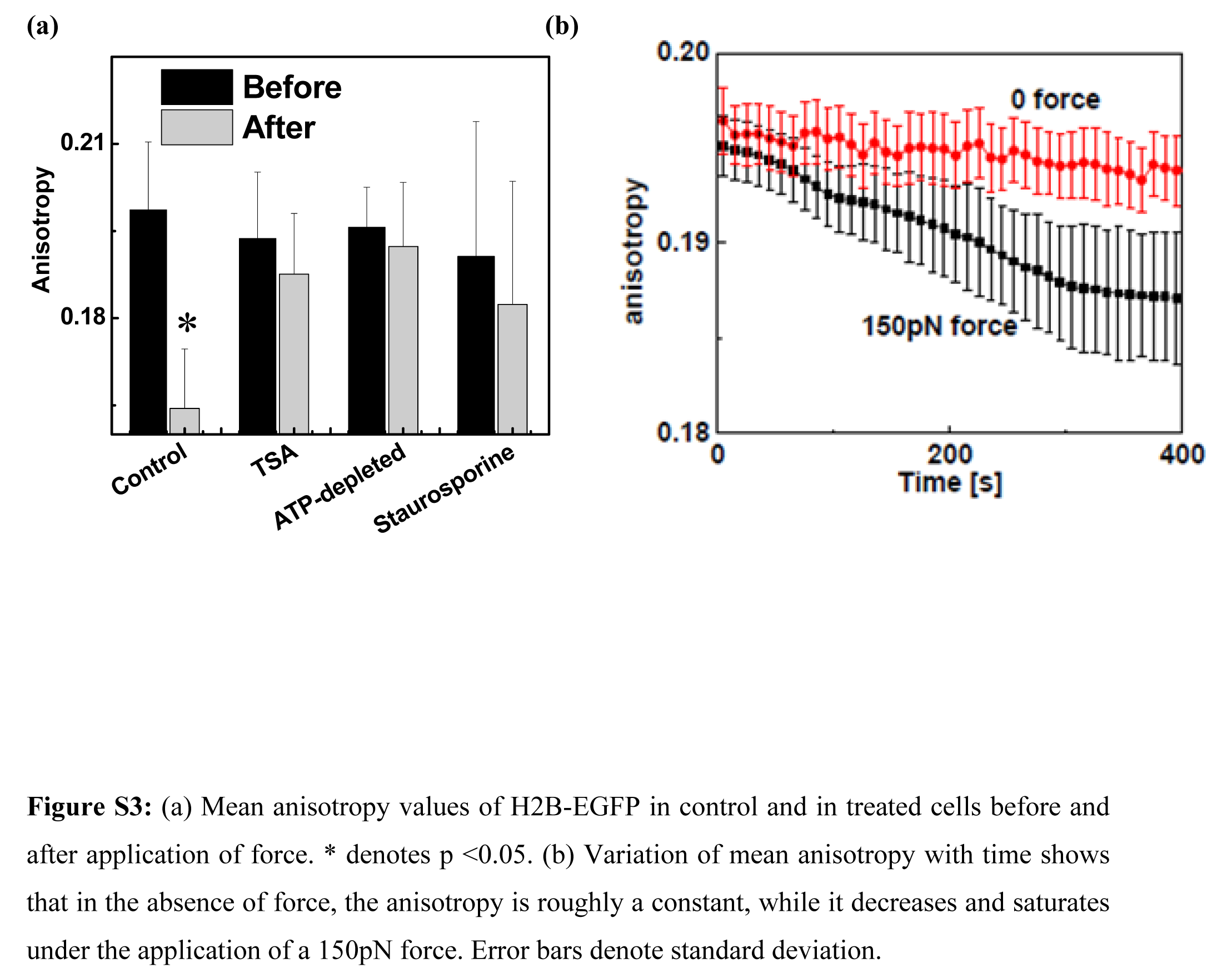

Supplement: Figure S3 — Chromatin packaging mapped as anisotropy of H2B-EGFP. (a) Mean anisotropy values of H2B-EGFP in control and in treated cells before and after application of force. * denotes . (b) Variation of mean anisotropy with time shows that in the absence of force, the anisotropy is roughly a constant, while it decreases and saturates under the application of a pN force. Error bars denote standard deviation. (TIF) [file pone.0045843.s003.tif]
